# Prevalence of gastro-esophageal reflux disease and its risk factors in a community-based population in southern India

**DOI:** 10.1186/s12876-016-0452-1

**Published:** 2016-03-15

**Authors:** Hai-Yun Wang, Kondarapassery Balakumaran Leena, Amelie Plymoth, Maria-Pia Hergens, Li Yin, Kotacherry Trivikrama Shenoy, Weimin Ye

**Affiliations:** Department of Medical Epidemiology and Biostatistics, Karolinska Institutet, Stockholm, 17177 Sweden; Population Health and Research Institute, Medical College PO, Trivandrum, Kerala 695011 India; Department of Gastroenterology, Sree Gokulam Medical College and Research Foundation, Trivandrum, 695607 India

**Keywords:** Gastro-esophageal reflux disease, Questionnaire, Prevalence, Risk factors

## Abstract

**Background:**

The prevalence of gastro-esophageal reflux disease (GERD) varies widely around the world. This study aimed to investigate the prevalence and risk factors of GERD in a general population of southern India.

**Methods:**

An interview-based observational study was carried out in southern India during 2010 and early 2011 using a GERD questionnaire (GerdQ). In total 1072 participants were enrolled using a multi-stage cluster sampling method. Presence of GERD was defined as a score of ≥ 8. Logistic regression models were used to derive odds ratios (ORs) with 95 % confidence intervals (CIs).

**Results:**

The prevalence of GERD was 22.2 % (238/1072) in southern India, and was more common among older subjects and men. Overweight and obese subjects had a dose-dependent increased risk of GERD, compared to those with body mass index less than 25 (multivariate-adjusted OR = 1.4, 95 % CI 1.0–2.0; OR = 2.3, 95 % CI 1.3–4.1, respectively). People residing in urban community were more vulnerable to GERD than those in rural community (multivariate-adjusted OR = 1.8, 95 % CI 1.3–2.5). Similarly, those with a lower educational level appeared to have an increased risk of GERD. Further, those with a habit of pan masala chewing were more likely to develop GERD compared with those abstained from the habit (multivariate-adjusted OR = 2.0, 95 % CI 1.2–3.2).

**Conclusions:**

GERD is highly prevalent in southern India. Increasing age and BMI, an urban environment, lower educational level, and pan masala chewing appear to be risk factors of GERD symptoms for the studied population.

## Background

Gastro-esophageal reflux disease (GERD) is one of the most common diseases in Europe and the United States, and affects severely the quality of life pertinent to such symptoms as heartburn and acid regurgitation [1]. The prevalence of GERD referring to those with symptoms at least once per week, varies greatly with ethnicity and geography: 18.1–27.8 % in North America, 8.8–25.9 % in Europe, and 2.5–7.8 % in East Asia, as estimated from 28 studies [2]. Recently, one study reported a GERD prevalence of 16.2 % among employees of a large hospital in Northern India [3]. However, there is a paucity of epidemiological data on the prevalence of GERD in general populations in Southern India. Previous population-based surveys conducted in Europe and North America have adopted a symptom-based approach (gastro-esophageal reflux disease questionnaire, GerdQ). This approach enables family practitioners and gastroenterologists to diagnose GERD accurately [4, 5]. In the present study, we sought to assess the prevalence of GERD and to explore its potential risk factors in a general population in Southern India using the GerdQ tool.

## Methods

### Study design

The pilot study was carried out during 2010 and early 2011, in the Trivandrum district of the southern Indian state of Kerala, which has the highest literacy, and a diverse population in relation to diet, ethnicity and religion. The study was community-based, and included the 12 blocks with 78 panchayats in the rural area, and the 81 wards in the urban corporation area. We adopted a multi-stage cluster sampling to enroll the study participants. Briefly, we randomly selected 3 blocks with 6 panchayats from these blocks in the first stage. For the six panchayats, we have 90 wards. Using a simple random sampling, 23 wards were selected. In the urban corporation area, we randomly selected 4 out of the 81 wards. In the second stage all dwelling houses were grouped into clusters of 7 houses each, using electoral roll, and 1072 participants were eventually recruited by a field team, through house-to-house survey. The response rate was 95 % in the rural, and 94 % in the urban areas. The study was approved by the ethical committee of Sree Gokulam Medical College, and the Regional Ethics Review Board in Stockholm. Informed consent was obtained from all participants.

### The symptoms of GERD and GerdQ

In this interview-based observational study, trained interviewers recorded symptoms of GERD using a recently developed and validated GERD questionnaire (GerdQ) [4, 6]. The GerdQ is a straightforward, self-administered and patient-oriented questionnaire with six items derived from three questionnaires [7–9], used to standardize the symptom-based diagnosis and evaluate treatment response in patients with GERD. In brief, six symptoms, four positive predictors (encompassing heartburn, regurgitation, sleep disturbance, and the use of additional over-to-counter (OTC) medication, using a four-graded scale (0–3)), and two negative predictors (encompassing pain or discomfort of the stomach and nausea with a reversed scale, 3–0), were used to evaluate the GERD frequency. This is considered to be a more objective and robust measurement of this essentially dichotomous symptom. The GerdQ score was calculated as the sum of each score of individual symptoms, giving a total score ranging from 0 to 18. According to previous validation studies, the optimal balance between sensitivity and specificity is achieved when using the cut-off value ≥ 8 [4], which was also used to define the presence of GERD in the current study.

### Measurement of other variables

Information was collected regarding age, sex, height, weight, residential areas, educational level, religion, and the habits of pan masala chewing and cigarette smoking. The study participants were categorized into three subgroups according to the different scales of body-mass index (BMI), determined by the weight in kilograms divided by the square of height in meters (kg/m^2^); BMI < 25 was referred as normal and served as reference, 25–29.9 as overweight and ≥30 as obese. The educational level was assessed by the years of schooling (low: 0–8 years; middle: 9–12 years; high: ≥13 years). The habits of pan masala chewing and cigarette smoking were dichotomized into never vs. ever.

### Statistical analysis

We calculated descriptive statistics of demographic characteristics for the study population, including age, sex, BMI, residential areas, educational level, religion, the status of pan masala chewing and cigarette smoking. Categorical and continuous variables are presented as frequencies and median (range), respectively. Using data from the questionnaire we calculated the prevalence of self-reported symptoms for GERD, overall or by above-mentioned variables. Logistic regression model was used to obtain odds ratio (ORs) with 95 % confidence intervals (CIs) for potential risk factors in relation to the presence of GERD. A p-value of < 0.05 is considered statistically significant. All statistical analyses were done by SAS statistical software, version 9.4 (SAS institute, Cary, NC, USA).

## Results

### Baseline characteristics

Of the 1072 individuals with a median age of 42 (range 20–86) years, 335 (31.3 %) were males and 737 (68.7 %) were females. Among them 709 (66.0 %) had a BMI < 25, and were considered as normal and the reference group; 298 (27.8 %) had a BMI between 25 and 29.9, considered as overweight, and 65 (6.1 %) had a BMI ≥ 30, considered as obese. Further, 731 (68.2 %) and 341 (31.8 %) were from rural and urban communities, respectively. Approximately one quarter of the participants reported having received 13 or more years of education. The participants belonged to the following religions: 891 (85.3 %) Hinduism, 108 (10.3 %) Muslim, and 46 (4.4 %) Christian. There were 101 (9.4 %) participants reporting a history of pan masala chewing, among whom 67 were men and 34 women. Smoking was more common than pan masala chewing (156 out of 1072, 14.6 %), and there were only three women among the ever smokers. Baseline characteristics of participants are displayed in Table 1.

**Table 1:** Basic characteristics of the study participants (*n* = 1072)

Characteristics		No. of subjects (%)
Total		1072
Age (y)	Median (range)	42 (20–86)
	20–29	173 (16.1)
	30–39	277 (25.8)
	40–49	275 (25.7)
	50–59	198 (18.5)
	≥60	149 (13.9)
Sex		
	Men	335 (31.3)
	Women	737 (68.7)
BMI (kg/m^2^)^a^		
	<25	709 (66.1)
	25–29.9	298 (27.8)
	≥30	65 (6.1)
	Unknown	2
Domicile		
	Rural	731 (68.2)
	Urban	341 (31.8)
Educational level (years)		
	Low (0–8)	344 (33.0)
	Middle (9–12)	446 (42.7)
	High (≥13)	254 (24.3)
	Unknown	28
Religion		
	Hinduism	891 (85.3)
	Muslim	108 (10.3)
	Christianity	46 (4.4)
	Unknown	27
Pan masala chewing		
	Never	970 (90.6)
	ever chewer	101 (9.4)
	Unknown	1
Cigarette smoking		
	Never	913 (85.4)
	ever smoker	156 (14.6)
	Unknown	3

^a^BMI, body-mass index

### The GERD prevalence and severity

In the present study, we surveyed the six items including the cardinal symptoms (heartburn and regurgitation) and atypical symptoms (nausea, night sleep disturbance, stomach pain, and additional medication) to define the presence of GERD. Individuals with GerdQ score of ≥ 8 were assigned to the GerdQ (+) group, whereas those with GerdQ score of < 8 were grouped into the GerdQ (−). The numbers of participants in the GerdQ (+) and GerdQ (−) groups were 238 (22.2 %) and 834 (77.8 %), respectively. Table 2 lists detailed information on the proportion of participants by frequency and severity for each item. In brief, the study participants reported ever having symptoms of the following: heartburn 283 (26.4 %); acid regurgitation 194 (18.1 %); night sleep disturbance 109 (10.2 %); use of additional OTC medication at least one day per week 99 (9.2 %).

**Table 2:** Frequency distribution of symptoms from six items included in GerdQ among 1072 participants

	Patient no. (%)			
Symptoms	0 day (never)	1 day (mild)	2–3 days (moderate)	4–7 days (severe)
Heartburn	789 (73.6)	90 (8.4)	113 (10.5)	80 (7.5)
Acid regurgitation	878 (81.9)	66 (6.2)	71 (6.6)	57 (5.3)
Stomach pain or discomfort	26 (2.4)	61 (5.7)	69 (6.4)	916 (85.5)
Nausea	15 (1.4)	46 (4.3)	61 (5.7)	950 (88.6)
Night sleep disturbance	963 (89.8)	53 (4.9)	42 (3.9)	14 (1.4)
Additional medication	973 (90.8)	30 (2.8)	41 (3.8)	28 (2.6)

### Potential risk factors of GERD

The associations of age, sex, BMI, domicile, educational level, religion, pan masala chewing, and cigarette smoking with GERD are presented in Table 3. In the univariate analyses, GERD presence was significantly associated with increasing age and BMI, living in an urban community, lower educational level, and ever pan masala chewing. In the further multivariate analysis, in which the variables listed in the table were mutually adjusted, results did not change materially.

**Table 3:** Variables associated with symptom-based GERD determined by GerdQ in the 1072 participants in south-western India

		GerdQ		Univariate	Mutually-adjusted
Variables		<8	≥8	Odds Ratio (95 % CI)	Odds Ratio (95 % CI)
Total		834	238		
Age (y)					
	20–29	157	16	1.0 (reference)	1.0 (reference)
	30–39	222	55	2.4 (1.3–4.4)	2.1 (1.1–3.9)
	40–49	215	60	2.7 (1.5–4.9)	2.2 (1.2–4.1)
	50–59	137	61	4.3 (2.4–7.9)	3.3 (1.7–6.3)
	≥60	103	46	4.4 (2.3–8.2)	3.0 (1.5–6.1)
Sex					
	Men	250	85	1.0 (reference)	1.0 (reference)
	Women	584	153	0.7 (0.5–1.0)	0.7 (0.5–1.1)
BMI (kg/m2)^a^					
	<25	570	139	1.0 (reference)	1.0 (reference)
	25–29.9	222	76	1.4 (1.0–1.9)	1.4 (1.0–2.0)
	≥30	42	23	2.2 (1.3–3.9)	2.3 (1.3–4.1)
Domicile					
	Rural	592	139	1.0 (reference)	1.0 (reference)
	Urban	242	99	1.7 (1.3–2.3)	1.8 (1.3–2.5)
Educational level					
	Low (0–8)	247	97	1.0 (reference)	1.0 (reference)
	Middle (9–12)	345	101	0.7 (0.5–1.0)	0.9 (0.6–1.3)
	High (≥13)	219	35	0.4 (0.3–0.6)	0.6 (0.4–1.0)
Religion					
	Hinduism	700	191	1.0 (reference)	1.0 (reference)
	Muslim	83	25	1.1 (0.7–1.8)	1.4 (0.8–2.3)
	Christian	30	16	1.9 (1.0–3.7)	1.6 (0.8–3.1)
Pan masala chewing					
	Never	768	202	1.0 (reference)	1.0 (reference)
	ever chewer	65	36	2.1 (1.4–3.3)	2.0 (1.2–3.2)
Cigarette smoking					
	Never	716	197	1.0 (reference)	1.0 (reference)
	ever smoker	116	40	1.3 (0.8–1.9)	0.7 (0.4–1.2)

^a^ BMI, body-mass index

## Discussion

This cross-sectional study has shown a high prevalence of GERD (22.2 %) in a general population residing in southern India, and significant associations between increasing age and BMI, urban environment, lower educational level, pan masala chewing and the presence of GERD. The prevalence of GERD in southern India is comparable with the range found in Western countries (8.8–27.8 %), but much higher than in East Asia [2]. In Asia the prevalence of GERD has gradually been increasing [10], which may be attributed to the growing economics and consequently change of lifestyle taking place in many Asian countries.

In our study, we observed a positive relationship of GERD with increasing age. A previous national multicenter study, conducted in China showed that the prevalence of reflux esophagitis increased with age [11]. However, the association between GERD and age is controversial. Several studies observed a positive relationship [12–14], whereas other studies have reported an inverse association [15], or a lack of relationship [3, 16–20].

Our finding of a positive association between increasing BMI and the risk of GERD is consistent with the results of many other studies [3, 12, 16, 20–24]. For example, a cross-sectional study showed that obesity increased the risk of GERD, partly explained by increasing esophageal acid exposure [20]. Another study indicated that the risk of GERD appeared to be linked directly to BMI, regardless of whether a person is of normal weight or overweight [24].

Not surprisingly, subjects living in an urban community have a consistent and higher risk of GERD compared to those living in a rural community. We speculate that subjects living in an urban area are susceptible to psychosocial factors contributing to the high prevalence of GERD as demonstrated in many previous studies [3, 14, 25, 26]. Our study also showed that subjects with higher educational level (≥13 years) had a lower prevalence of GERD. This is in line with findings observed in Albania [12] and another study among monozygotic twins, which showed that lower educational level may increase the risk of GERD in women but not in men [21].

Interestingly, in the present study we did not find an association between cigarette smoking and the risk of GERD, as shown in previous studies conducted in Sweden, Spain and the United States [16, 19, 27]. To the best of our knowledge, we are the first to show the importance of pan masala chewing for the development of GERD symptoms. Confounding by other variables does not completely explain the observed association, as the strength of the association remained unchanged after multivariate adjustment. However, the underlying mechanism for the observed pan-masala-chewing-GERD association is still unclear, although we speculate that certain additives of pan masala may reduce the pressure of lower esophageal sphincter during chewing. The ingredients of pan masala vary widely. Pan masala is a form of chewable tobacco commonly used in India, which is a mixture of betel leaf with areca nut, tobacco and lime, and it may also contain Katha paste in some south Asian populations. Two previous studies conducted in India [28] and Pakistan [29] looked at different types of pan masala, and showed consistently that pan masala with or without tobacco was a strong risk factor for oral cancer. Moreover, several investigations have experimentally demonstrated that lifetime feeding of pan masala induces adenoma of several organs and neoplastic lesions in the liver, stomach and lung [30]. If the association between pan masala chewing and GERD can be confirmed, we would expect to observe a positive association between pan masala chewing and esophageal adenocarcinoma, as GERD is the most important risk factor for this malignancy [31].

The main limitation of our study is that it is a pilot study and a larger sample size is required to consolidate the observed associations between potential risk factors and the presence of GERD. Admittedly, a prospective cohort design would have been more powerful than the used cross-sectional design in establishing a causal relationship between observed risk factors and the presence of GERD. Another limitation is that we lacked data of 24-h pH monitoring and had to rely on questionnaire data only to define GERD. However, several published studies have shown the validity and reliability of GerdQ, in identifying cases of GERD [4, 6–9]. Moreover, the symptoms have been correlated with objective complications of GERD, such as esophagitis and esophageal adenocarcinoma [19, 32].

## Conclusions

In conclusion, this cross-sectional study shows a high prevalence of GERD in a general population in southern India. The risk factors predisposing for GERD in the study population include increasing age and BMI, living in urban area, lower educational level, and pan masala chewing.

## Availability of data and materials

All the data supporting our findings is contained within the manuscript.
